# Antiseptic Surgery of the Mouth and Face

**Published:** 1900-12-15

**Authors:** W. H. G. Logan

**Affiliations:** Chicago, Ills.


### ANTISEPTIC SURGERY OF THE MOUTH AND FACE.

BY W. H. G. LOGAN, D.D S , CHICAGO, ILLS.

Read before the National Dental Association, July 10, 19C0.

If you please, let us first take under consideration the steps necessary to gain and maintain an aseptic wound about the face. The part needing surgical interference should be first shaved, then scrubbed with a stiff nail brush and green soap; then cleansed with a 2 percent carbolic acid solution, and lastly bathed freely with absolute alcohol; the patient being previously anesthetized and hair so bandaged that the surgeon can not get his fingers or instruments in contact with the part, and as a result infect the wound. If during the operation the patient vomits, the wound and the field about should at once be covered with gauze, which is not removed until the parts beyond the surgical field have been made aseptic again. The nausea subsiding, the protecting gauze is removed and the operation finished. The wound is irrigated with a 1 percent carbolic acid solution, and followed by a saturated solution of boracic acid ; care is taken that all free pieces of tissue, bits of cotton, or tents of gauze are carried away during the process of irrigation. Hemorrhage in this field can, as a rule, be best controlled by torsion. In case this fails ligate with catgut. Never use a powerful styptic to control hemorrhage which occurs in an aseptic wound ; it will prevent union in the parts by first intention. The employment of tampons passed from boiling water to the wound will control the hemorrhage you have occasion to treat about the mouth and face very satisfactorily in a majority of cases, with the pleasant after-result of but slight wound inflammation. You may now close the wound by the employment of horsehair or silk and catgut sutures, the catgut only to be employed where the wound is very deep. I do not believe it wise to rely upon buried sutures alone in the treatment of wounds about the face, but always use a few horsehair or silk sutures to insure constant coaptation of the edges. Care should be taken that these sutures are not drawn too tight, for the result would be stitch abscesses.

Let us now pass to the dressing of this aseptic wound of the face. Questions of importance are: Should it be a moist or dry dressing? How secured to the part? and period of time dressing is to be employed. Answers: A dry dressing, and secured to the part by the use of flexible collodion to the dried tissue. This dressing is kept in position from five to twelve days, dependent upon the tension about the wound. To prepare the surgical field for its dressing after the sutures are in position, dry the parts and dust thickly over the sutured line with powdered boracic acid, which is then moistened with alcohol. The powdered boracic acid will, as the alcohol evaporates, practically hermetically seal the exposed edges of our aseptic wound. Now place a few layers of gauze, to be held secure by bandaging where the part will allow. More frequently, however, it will be found necessary to place a fold of absorbent cotton over the layers of gauze, and secure this to the dried tissue by the means of flexible collodion and adhesive plaster strips. The dressing is not to be disturbed without good cause until the stitches are to be removed, which is from the fifth to the eighth day, after which the surface is again prepared as before and a similar dressing replaced for a period of four days, to allow of closure by granulation of the openings caused by the removal of the sutures. Always employ the dry dressing for all aseptic wounds of the face, for the reasons that with a dry dressing you can practically hermetically seal the wound by the procedure spoken of; secondly, because the bacteria brought in contact with the wound by the air can not propagate without moisture.

We shall now pass to the consideration of the probability and possibility of gaining and maintaining such a condition of a surgical wound in the oral cavity after an operation that infection will not take place from the pyogenic bacteria ever present in the fluids of the oral cavity. Although a quarter of a century has elapsed since the value of aseptic and antiseptic methods of general surgery were brought forward, the benefits derived from following these principles in the treatment of external wounds can be found detailed in all text-books of surgery of recent date, while the methods and procedure necessary to gain and maintain a condition in and about a surgical wound of the mouth are dismissed with but a line or so, if even spoken of.

What precautions should be taken to prevent the proliferation of the bacteria which are present in the saliva that bathes the wound constantly? This fluid is ordinarily alkaline or neutral, a perfect medium for the growth of the streptococci and staphylococci, which are the main pus-producing germs found, as a rule, in this field. Can not the proliferation and activity of these pyogenic germs and their dire effects be best prevented in this field by changing the normal secretions from alkaline, or by producing a condition which will prevent the development of these bacteria? If we can by any harmless method maintain a mild acidity of the fluids which bathe the surgical wound of the mouth without toxic effect, will we not gain asepsis of the oral cavity by the presence of free oxygen upon the wound's surface?

With the hope of changing the alkaline condition to one of acidity, the powdered acetate of potassium was dusted on the surface of wounds made in the mouths of two dogs for experimental work, with the idea that the salt would decompose and thereby leave liberated acetic acid on the wound surface. The wounds thus treated healed without undue inflammation. No infection occurred, and the wounds looked fresh and clean at each application. The wound of dog No. 1 healed in nine days; of dog No. 2 in ten days.

We experimented also on two dogs with similar wounds in the cheek with the bacilli acidi lacti. The wounds were dusted with this bacillus for the purpose of having ever present over the wound surface a slight acid secretion. Sugar was used with the bacteria merely to dilute it. These wounds healed a trifle slower than those upon which the acetate of potassium was employed, but healed in eleven days. The discharge of secretion was rather pronounced, yet no infection took place. The wound was ever fresh and clean at the various applications.

In the next two dogs, under experiment with like wounds, oxychlorin was dusted over the part with the hope of having oxygen liberated constantly on the wound's surface, which would prevent the development of bacteria. The oxychlorin treatment in case No. 1 did not prevent the formation of pus; a slide made during the second day showed the presence of streptococci and staphylococci. Yet the infection lasted only two days, while the wound healed in ten days without the formation of scar tissue. The second dog's wound, from like treatment, healed in nine days, and showed a beautiful clean surface at all times. The various wounds were treated three times daily.

This experimental work was carried further by again making like wounds in length and depth in the mouths of these dogs and infecting them with a virulent culture of staphylococcus pyogenes aureus. The wounds were not treated until the next day, to allow perfect opportunity for the growth of the bacteria.

The wounds under the treatment with oxychlorin showed on the second day of treatment inflammation and a great quantity of pus, while the slide made showed the presence of staphylococci The inflammation did not cease increasing until the ninth day of treatment had passed, though the pus began to decrease at the end of the seventh day, ceasing in ten days more. The dogs under oxychlorin treatment had to be fed upon a bread and milk diet for twelve days, as a result of the great inflammation in the cheek tissue.

In the cases where potassium acetate was employed the inflammation and infection increased for a period of five days only, yet the slides made showed the same infection as in the preceding case. The inflammation and pus secretion practically ended after eleven days; infection controlled in fourteen days.

Reviewing the experiments where the wounds were infected with the same virulent culture of staphylococcus pyogenes aureus, the slides made the following day showed the same infection. These wounds were dusted on the following day with another culture of bacteria, using the bacilli acidi lacti and sugar. Slight inflammation about the wounds' edges appeared during the fourth day; the afternoon of the fifth day the inflammation began to increase, and continued to increase slowly for three days. When eight days of treatment had passed the inflammation and infection were rapidly subsiding, and on the morning of the tenth day a fresh, clean surface was presented, with but slight oozing of secretions; and slides made on the evening of the eleventh day of the wound that had been dusted with the lactic acid producing bacillus showed the wound to be free from infection and healing kindly.

The wounds which were infected with the pus-producing germs and treated by oxychlorin, potassium, acetate, and the lactic acid-producing bacteria (bacilli acidi lacti), showed, in brief, the following conditions:

Under oxychlorin the infection was controlled in seventeen days; pronounced inflammation present, fourteen days; pronounced swelling present, twelve days; bread and milk diet, twelve days.

Under potassium acetate, infection was controlled in fourteen days; pronounced inflammation present, six days; pronounced swelling present, four days; soft food as diet, four days.

Under bacilli acidi lacti and sugar, infection controlled in eleven days; pronounced inflammation not present; pronounced swelling not present; regular diet throughout.

We are led to assume that the reason that wounds under treatment with the lactic acid-producing bacillus and acetate of potassium did so well was because we were able by this procedure to create and maintain more constantly over the wound surface a slight acid condition instead of an alkaline or neutral one.

In closing, I wish to say I shall carry this experimental work further on similar grounds, including the treatment of the pus pockets and suppurative conditions which we frequently find about the roots of teeth, using potassium acetate and the lactic acid-producing bacillus to see if we can not control more quickly the infection in these parts. —*Dental Cosmos.*

